# Proteome and miRNAs Expression in Medication-Related Osteonecrosis of the Jaw

**DOI:** 10.3390/ijms27115141

**Published:** 2026-06-05

**Authors:** Alessandro Allegra, Rossana De Salvo, Antonia Marcianò, Francesca Polito, Fabio Stagno, Alfonso Carleo, Michele Costanzo, Marianna Caterino, Marco Ragusa, Laura Licitri, Selene Francesca Anna Drago, Irene Gasparo, Giuseppe Alberti, Marieme Khouyyi, Enrico Nastro Siniscalchi, Giacomo Oteri, Luca Bini, Vincenzo Macaione, Laura Bianchi, M’hammed Aguennouz

**Affiliations:** 1 Department of Human Pathology in Adulthood and Childhood “Gaetano Barresi”, University of Messina, 98125 Messina, Italy; alessandro.allegra@unime.it (A.A.); stagnof@unime.it (F.S.); giuseppe.alberti@unime.it (G.A.); 2 Laboratory of Functional Proteomics, Department of Life Sciences, University of Siena, 53100 Siena, Italy; rossana.desalvo@unisi.it (R.D.S.); luca.bini@unisi.it (L.B.); laura.bianchi@unisi.it (L.B.); 3 Department of Biomedical and Dental Sciences, Morphological and Functional Images, University of Messina, 98125 Messina, Italy; antonia.marciano@unime.it (A.M.); marieme.kouyyi@unime.it (M.K.); giacomo.oteri@unime.it (G.O.); 4 Department of Clinical and Experimental Medicine, University of Messina, 98125 Messina, Italy; laura.licitri@unime.it (L.L.); irene.gasparo@unime.it (I.G.); vincenzo.macaione@unime.it (V.M.); aguenoz@unime.it (M.A.); 5 Laboratory of Molecular Medicine and Genomics, Department of Medicine, Surgery and Dentistry “Scuola Medica Salernitana”, University of Salerno, 84081 Baronissi, Italy; alfonso.carleo@unisa.it; 6 Department of Molecular Medicine and Medical Biotechnology, University of Naples Federico II, 80131 Naples, Italy; michele.costanzo@unina.it (M.C.); marianna.caterino@unina.it (M.C.); 7 CEINGE–Biotecnologie Avanzate Franco Salvatore, 80145 Naples, Italy; 8 Department of Biomedical and Biotechnological Sciences, University of Catania, 95123 Catania, Italy; marco.ragusa@unict.it; 9 Department of Chemical, Biological, Pharmaceutical and Environmental Sciences, University of Messina, 98166 Messina, Italy; sedrago@unime.it; 10 Department of Medicine and Surgery, University of Enna Kore, 94100 Enna, Italy; enrico.siniscalchi@unikore.it

**Keywords:** medication-related osteonecrosis of the jaw, multi-omics profiling, necroptosis, Runx1/Runx2, desmosome, pigment epithelium-derived factor, desmoglein-1, osteoblast signaling, osteoclast regulation, angiogenesis, matrix mineralization, inflammation

## Abstract

Medication-related osteonecrosis of the jaw (MRONJ) is a complex condition associated with the use of antiresorptive drugs, such as bisphosphonates and denosumab. The condition is characterized by the presence of exposed bone in the maxillofacial region that fails to heal. MRONJ remains highly intractable, as its pathogenic mechanisms are not yet fully understood. It is therefore essential to elucidate the molecular mechanisms underlying the disease. MiRNA expression analysis and proteomic studies were performed on a selected cohort of patients with MRONJ on jawbone tissue, using qRT-PCR and 2D electrophoresis followed by mass spectrometry. MiRNAs and proteomics data validation was carried out by Western blot analysis of differentially expressed proteins highlighted by a proteome study and predicted targets of differentially expressed miRNAs. Nineteen miRNAs were overexpressed and two downregulated in jawbone tissue from all MRONJ patients. Notably, five of these dysregulated miRNAs are involved in the regulation of angiogenesis and desmosome functions, suggesting a potential link to the molecular alterations observed at the protein level. Proteomic analysis revealed decreased concentrations of the pigment epithelium-derived factor, and of desmoglein-1, a desmosomal cadherin. Validation analysis confirmed the dysregulation of pathways involved in bone remodeling and necroptosis. The pathophysiology of MRONJ arises from a complex interplay of factors, including impaired bone remodeling, affected angiogenesis, and altered cell adhesion and differentiation mechanisms, ultimately leading to necroptosis. Through proteomic analysis and validation of miRNA expression, our study proposes specific molecular alteration in MRONJ-compromised bone tissue, involving desmosomal component imbalance and angiogenesis inhibition.

## 1. Introduction

An adverse drug reaction known as osteonecrosis of the jaw (ONJ) may occur in patients who have never undergone radiotherapy when receiving antiresorptive and antiangiogenic drugs [1,2].

It is characterized by the gradual loss and decomposition of bone in the mandible or maxilla. With respect to clinical features, previous research has demonstrated that the average age of patients at risk is 65.3 years, most of them are female, and the mandible is the most affected jawbone [3].

The increased prevalence of medication-related osteonecrosis of the jaw (MRONJ) is mostly due to the use of antiresorptive drugs, such as bisphosphonate and denosumab. Given the wide global distribution of neoplastic disorders, the annual total number of new cases of MRONJ is fairly considerable. According to a Japanese study, the yearly incidence of low-dose MRONJ was 132.5 per 100,000, and the annual incidence of high-dose MRONJ was 2305.8 per 100,000. When comparing patients with osteoporosis who took low-dose antiresorptive drugs to cancer patients who took high-dose agents, the incidence ratio was 23.6 for the former group and 420.6 for the latter. The incidence of MRONJ rose between 2016 and 2020, while the incidence of high-dose MRONJ declined—albeit not significantly [4].

Many explanations have been proposed for the origin of MRONJ, including immunological dysfunction, angiogenesis inhibition, osteoclastic bone remodeling and resorption inhibition, inflammation and infection, and soft tissue toxicity [5,6,7]. The role of the vascular endothelial growth factor (VEGF) in the context of MRONJ is also relevant. Bisphosphonates have been shown to inhibit circulating levels of VEGF, a key angiogenic factor, which may contribute to the impaired healing observed in patients receiving these therapies. This inhibition of VEGF not only alters angiogenesis but also compromises the overall bone remodeling process, further exacerbating the risk of osteonecrosis [8].

MRONJ remains highly intractable, as neither a pathogenic mechanism nor a standardized therapy have been established [9]. Moreover, no clinically meaningful biomarker for MRONJ has been identified to date, possibly due to an incomplete understanding of its underlying mechanisms.

Recent studies have highlighted the role of miRNAs as potential biomarkers and therapeutic targets in the context of MRONJ. For example, altered miRNA expression profiles have been observed in patients with MRONJ, suggesting that these small noncoding RNAs may play crucial roles in disease pathogenesis. Specifically, miRNAs are known to regulate gene expression at the post-transcriptional level, influencing various cellular processes, including inflammation and bone metabolism, which are critical in the development of MRONJ [10,11]. Moreover, a circulating miRNA panel has been proposed as a novel diagnostic tool for MRONJ, indicating that specific miRNA signatures could help in identifying patients at risk for developing this condition [12].

While transcriptomic analysis provides insights into gene expression, it is not able to explain the complex protein functional networks that govern cellular functions in physiological and pathological conditions. Consequently, the aim of the present study is to study proteome and miRNAs expression in the bone tissue adjacent to the lesion in patients with MRONJ as well as in apparently healthy controls and investigate whether differential protein analysis can provide insights into the pathogenetic mechanisms of the disease.

This integrated approach could provide new insights into the aberrant biomolecular processes underlying MRONJ, shedding light on the mechanisms that drive the disease onset and progression.

## 2. Results

### 2.1. miRNA Expression

A miRNA expression profile was performed on bone extracts from patients and healthy controls (CTRL) using single TaqMan assays. According to the cut-off criteria (fold change <2 downregulation and fold change >2.0 upregulation), results obtained evidenced a total of 21 differentially expressed miRNAs; of them, 19 were upregulated and two were downregulated (Table 1).

The enrichment analysis of biological processes related to targets of these miRNAs, carried out with DIANA-miRPath v4.0, revealed the involvement of these miRNAs in various signaling pathways. Analyzing these pathways, our attention has focused on three miRNAs (miR-30b-5p, miR-204-5p, miR-222-3p) whose interactive action inhibits pathways regulating bone development with runt-related transcription factor 1 and 2 (Runx1 and Runx2) and nuclear factor of activated T-cells 5 (NFAT5) as transcription factors.

**Figure 1:**
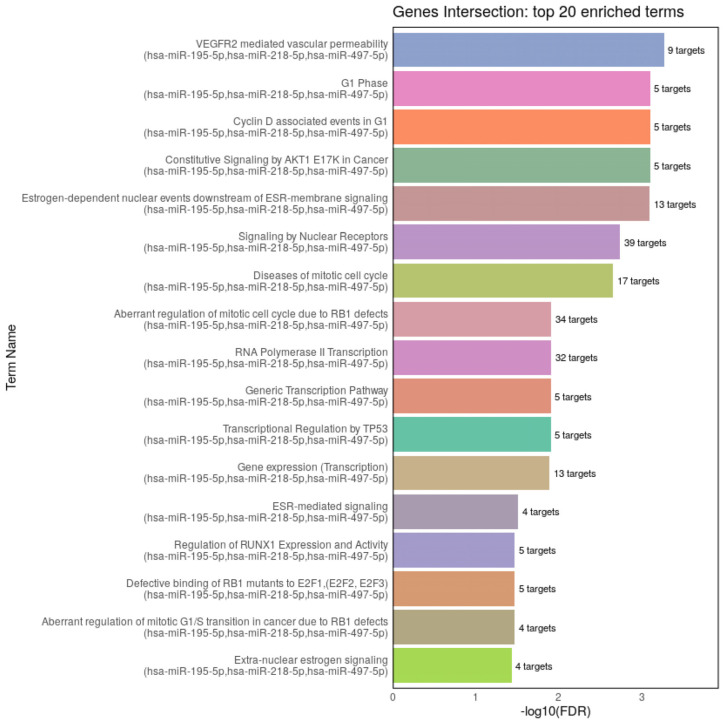
The most strongly enriched biological processes related to gene intersection of miR 30b-5p, miR-204-5p, miR-222-3p.

In addition, analysis of miRNAs targeting TNFAIP3 using miRTarBase (GeneCards.org, https://mirtarbase.cuhk.edu.cn) identified four miRNAs from our list: miR-125a-3p, miR-204, miR-211, and miR-505-5p. According to the KEGG pathway analysis (Figure 1), TNFAIP3 is directly involved in necroptosis regulation. These miRNAs target and inhibit the TNFAIP3-regulated pathway, leading to RIPK1 activation and triggering necroptosis.

KEEG Bioinformatic analysis of upregulated miRNAs found in MRONJ patients showed that the target genes were involved in a variety of cellular functions. The top significantly related GO terms are listed in Table 2.

**Figure 2:**
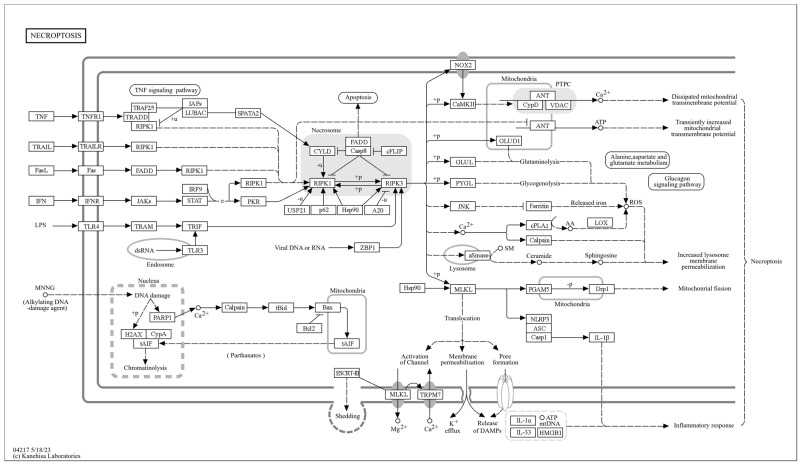
KEGG. map04217: Necroptosis. Kyoto Encyclopedia of Genes and Genomes, from https://www.kegg.jp/entry/map04217 (accessed on 15 April 2026).

The KEGG analysis showed the involvement of the upregulated target genes in important pathways such as the FoxO signaling pathway, TNFSF10-RIPK1/3 signaling pathway Mitophagy, and the mitogen-PI3K-Akt signaling pathway; combining these metabolic pathways leads to a major pathway of necroptosis, as seen in Table 3 and Figure 2.

### 2.2. Proteomics Investigation of Bone Samples from the Jaw

Owing to the significant bone degradation with extensive nonspecific proteolysis in ONJ, as evidenced by preliminary analyses (Figure S1, Supplementary Materials), our proteomics study was conducted on bone tissues adjacent to the area visually affected by advanced osteonecrosis. Furthermore, considering the substantial patient-dependent heterogeneity of the excised samples, primarily due to variations in blood perfusion and in the severity of bone disruption, the three samples from patients with ONJ were selected on the basis of relative morphological homogeneity. Similarly, three samples from apparently healthy individuals, not presenting the condition, were also selected.

**Figure 3:**
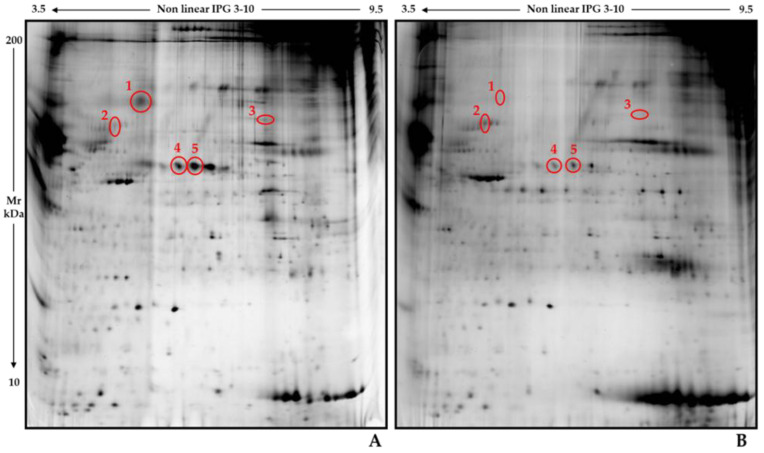
Representative 2DE protein patterns of jaw bone from a control subject (**A**) and a MRONJ patient (**B**). Red circles and numbers indicate differentially abundant protein spots detected by comparing the two conditions (FC ≥ 2; FDR ≤ 0.05).

Sample selection was accurate, but the analyses revealed substantial individual-dependent heterogeneity probably due to therapy intraindividual reaction, thus reducing to five the number of significantly different protein spots between the two conditions under investigation (Figure 3; analyzed gel images are provided in Figure S1 of the Supplementary Materials).

These differences, however, are highly significant due to the application of rigorous gel image analysis, statistical evaluations, and filtering criteria based on %Vol ratios ≥2.

**Figure 4:**
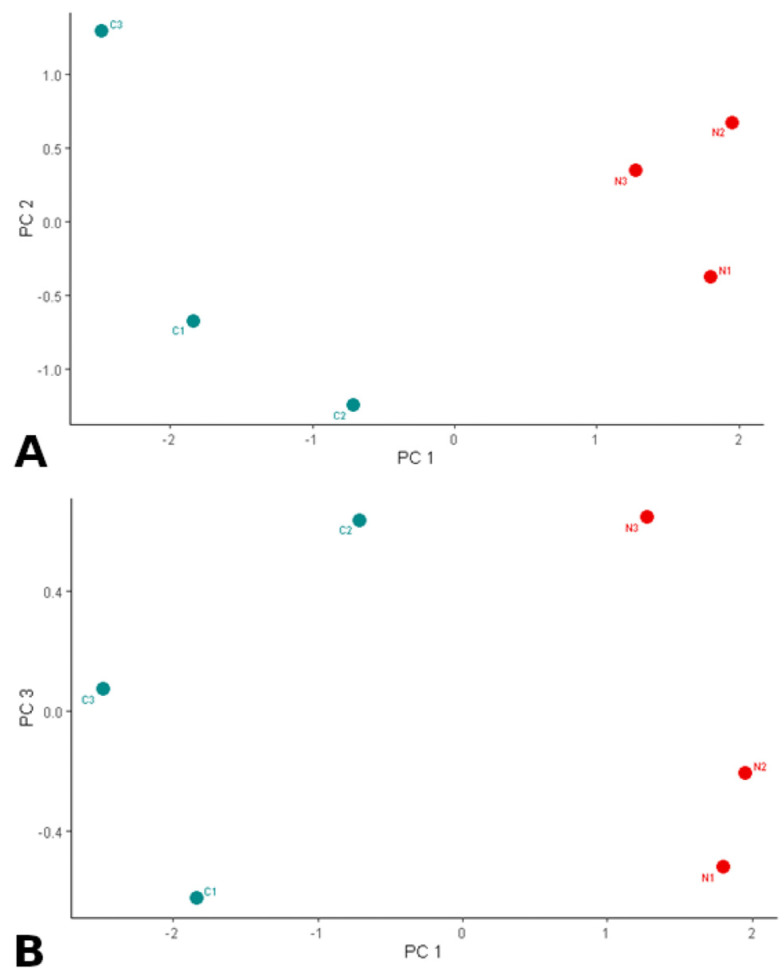
Principal component analysis (PCA) performed on the %Vols of spot differences detected between jaw samples from control subjects and ONJ patients. Plots highlight spatial distribution of the six analyzed bone samples—three from controls (cyan symbols; C1–3) and three from ONJ cases (red symbols; N1–3)—along PC1 (75% of variance) and PC2 (17.6% of variance) (**A**) and PC1 and PC3 (6.2% of variance) (**B**).

According to the variance analysis, these differences allowed the six analyzed samples to be distinctly grouped into two clusters: controls and patients. Specifically, principal component analysis (PCA) revealed that the separation between controls and patients was primarily driven by PC1, which accounted for 75% of the total variance. The distribution of controls in the PC1/PC2 plot depends on both PC1 and PC2, with the latter explaining 17.6% of the variance. In contrast, the patient cluster exhibited reduced intra-cluster heterogeneity in relation to both PC1 and PC2, while its distribution along PC3 was comparable to that of the controls. However, PC3 explained only 6.2% of the variance (Figure 4).

The tighter clustering observed in patients than in controls underlined that the five identified differences distinctly characterize the pathological condition, independently of individual variability. These may therefore be considered relevant ONJ biomarkers.

**Figure 5:**
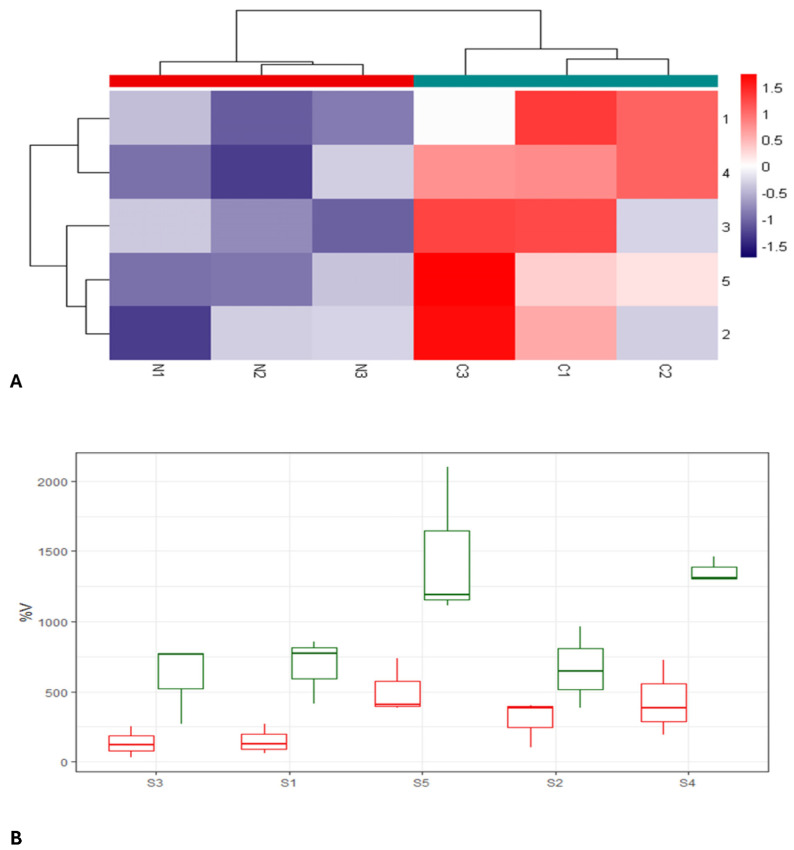
Heatmap visualizing the relative abundances of the five significant differences detected among healthy (C1–3) and osteonecrosis-affected (N1–3) jaws. Each row corresponds to a spot, whereas each column represents a sample. The row numbers, reported on the right of the matrix, match those reported on gel images from Figure 2. The color gradient spans from red to blue, indicating higher or lower signal intensities, respectively. The samples clearly clustered into two groups, as highlighted by the horizontal dendrogram: the control (cyan bar) and the patient clusters (red bar) (**A**). Box plot of the five significant differences (**B**), the spot numbers (S1–S5) match those from Figure 2 and the colours those of the heatmap bar.

The subsequent Pearson correlation analysis, followed by the application of Ward’s method for hierarchical clustering via Euclidean distance, minimized variance within each cluster and facilitated the construction of a dendrogram. The correlation matrix was then converted into a distance matrix for clustering, and the resulting dendrogram was combined with the heatmap of the correlation matrix. This provided a comprehensive visual representation, highlighting correlation patterns and the cluster structure of similar variables. The heatmap colors represent the intensity of the numerical values in the distance matrix derived from the Pearson correlation matrix, following a continuous scale from −1.5 (perfect negative correlation; intense blue) to +1.5 (perfect positive correlation; intense red). Overall, the resulting image reported in Figure 5A stresses, as PCA did, sample clustering into two distinct sample groups, in reason of their %Vol values, corresponding to the control cluster (cyan bar) and to the ONJ cluster (red bar). Interestingly, all the five significant protein differences we detected resulted down-regulated in the disease and, despite the intra-cluster variance, the abundance-decrease trend, characterizing the disease state, is clearly evident in the box plot shown in Figure 5B.

### 2.3. LC–MS/MS Analysis

Tandem MS analysis allowed the identification of several proteins differentially resolved into the processed spots. Notably, we identified pigment epithelium-derived factor (PEDF) and desmosomal cadherins, desmoglein-1, desmoplakin and desmocollin-1, which are all involved in regulating osteogenic differentiation and proliferation (Table 4).

### 2.4. miRN-Omics and Proteomics Data Validation

miRNAs and proteomics data validation was carried out by Western blot analysis of differentially expressed proteins highlighted by the proteome study and predicted targets of differentially expressed miRNAs, analyzing the expression levels of the pigment epithelium-derived factor (PEDF), desmoglein-1 (DSG1) and desmocollin-1 (DSC1), RUNX1, RUNX2, TNFAIP3, RIP3 and VEGF, involved in regulation of osteogenic differentiation, proliferation and necroptosis.

**Figure 6:**
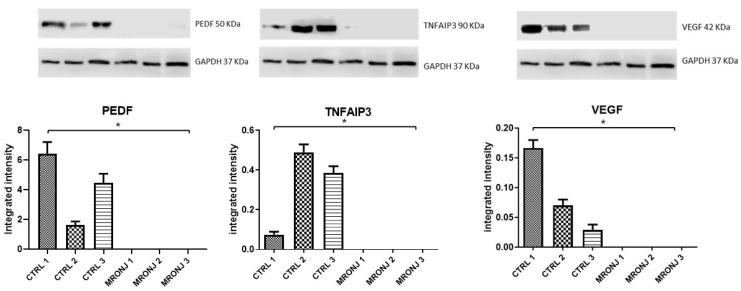
miRNAs and proteomics data validation by western blot analysis from three control subjects and three MRONJ patients. Quantification was performed by densitometric analysis with the LICOR imaging system, and results were expressed as relative intensity normalized to GAPDH, used as an endogenous control. The upper panel showed the representative autoradiography highlights expression of PEDF, TNFAIP3, VEGF and GAPDH expression. The lower panel showed the quantitative data representing the mean ± SD (error bars) of three indipendent replicates. * = *p* < 0.05 vs. CTRL.

**Figure 7:**
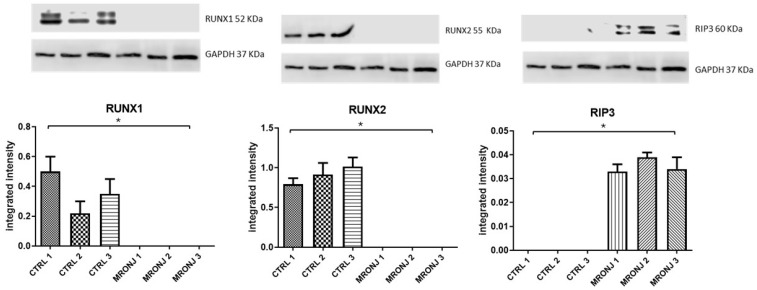
miRNAs and proteomics data validation by western blot analysis from three control subjects and three MRONJ patients. Quantification was performed by densitometric analysis with the LICOR imaging system, and results were expressed as relative intensity normalized to GAPDH, used as an endogenous control. The upper panel showed the representative autoradiography highlights expression of RUNX1, RUNX2, RIP3 and GAPDH expression. The lower panel showed the quantitative data representing the mean ± SD (error bars) of three indipendent replicates. * = *p* < 0.05 vs. CTRL.

**Figure 8:**
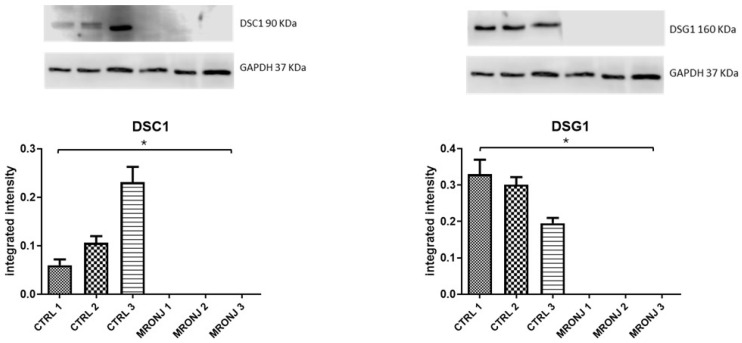
miRNAs and proteomics data validation by western blot analysis from three control subjects and three MRONJ patients. Quantification was performed by densitometric analysis with the LICOR imaging system, and results were expressed as relative intensity normalized to GAPDH, used as an endogenous control. The upper panel showed the representative autoradiography highlights expression of DSC1, DSG1 and GAPDH expression. The lower panel showed the quantitative data representing the mean ± SD (error bars) of three indipendent replicates. * = *p* < 0.05 vs. CTRL.

Results obtained confirm the dysregulation of these proteins in MRONJ with respect to healthy controls (Figure 6, Figure 7 and Figure 8). In more detail, we found a reduced expression of PEDF, VEGF and TNFAIP3 (Figure 6), RUNX 1, RUNX 2 and RIP3 (Figure 7), DSG and DSC1 (Figure 8) in MRONJ compared to those observed in healthy bones, while an upregulation of RIP3 was observed in MRONJ samples (Figure 7).

## 3. Discussion

In the present study, we used a combined approach of proteomics and miRNA analysis for the identification of biomarkers potentially involved in the development of MRONJ.

Proteomic analysis identified some proteins with reduced abundance in MRONJ samples, PEDF, desmoglein-1, desmoplakin, and desmocollin-1, all of which are likewise implicated in osteogenic processes, while complementary miRNAs analysis evidenced the upregulation of different miRNAs in the jawbone tissue of MRONJ patients involved in bone development and necroptosis. These molecular alterations collectively point toward a disruption in the transcriptional and structural jaw bone machinery governing osteoblast differentiation and bone matrix maintenance.

More specifically, target prediction revealed that three upregulated miRNAs (i.e., miR-30b-5p, miR-204-5p, miR-222-3p) may contribute to the regulation of essential osteogenic transcription factors, namely Runx1, Runx2, and the nuclear factor of activated T-cells 5 (NFAT5).

The Runt-related transcription factors, particularly Runx1 and Runx2, are involved in bone development and homeostasis. Runx2 is widely recognized as the master regulator of osteoblast differentiation and bone formation. It orchestrates the transcriptional program required for the commitment of mesenchymal stem cells to the osteoblast lineage, by promoting the expression of critical bone matrix proteins such as collagen type I, osteopontin, and osteocalcin [13]. This regulatory role is critical during various stages of skeletal development, including endochondral ossification, where Runx2 facilitates the transition of chondrocytes into osteoblasts, thereby enabling bone mineralization [14]. Additionally, Runx1 participates in osteoblast proliferation and differentiation, and accumulating evidence suggests that it may partially compensate for Runx2 deficiency, thereby implying functional overlap between these transcription factors in bone development [15,16].

In our study, validation analysis confirmed a marked decrease in Runx1 and Runx2 in the MRONJ jawbone, therefore suggesting a possible functional association between the upregulation of miR-30b-5p, miR-204-5p, and miR-222-3p and altered osteogenic signaling, which could potentially compromise bone regeneration and the healing processes in MRONJ.

Alongside the miRNAs described above, we detected three other upregulated miRNAs in MRONJ tissue samples, i.e., miR-125a-3p, miR-211, and miR-505-5p, which, together with miR-204-5p, are known to target tumor necrosis factor alpha-induced protein 3 coding gene (TNFAIP3), a pivotal modulator in inflammatory processes and bone formation.

TNFAIP3 functions as a negative regulator of NF-κB signaling, thereby playing a central role in limiting inflammation and preserving tissue homeostasis. It exerts both enzymatic (ubiquitin editing) and non-enzymatic functions to control NF-κB activation and downstream effects. Moreover, TNFAIP3 acts as a cytoprotective factor, capable of inhibiting both apoptosis and necroptosis [17], the latter being a form of regulated necrosis mediated by receptor-interacting protein kinases (RIPKs) that offers an alternative route for cell demise when apoptosis is defective and that actively promotes and amplifies inflammation [18]. Our results showed decreased TNFAIP3 levels and concomitant RIP3 upregulation in MRONJ patients, suggesting a potential interplay between altered miRNA expression and TNFAIP3/RIP3 signaling in the pathogenesis of the disease, and raising the possibility that necroptosis may contribute to MRONJ development. MiR-125a-3p, miR-211, miR-204-5p, and miR-505-5p, through TNFAIP3 downregulation and RIP3 upregulation, may hence contribute to MRONJ pathogenesis by increasing inflammation, reducing cytoprotective signaling and facilitating necroptosis-mediated tissue injury, thereby exacerbating tissue damage and disrupting bone homeostasis [19]. In this altered regulatory context, proteomic analysis revealed the decreased presence of PEDF in MRONJ that might further compromise bone physiology, given its multifaceted role in osteoblast differentiation, matrix mineralization, and overall bone integrity and strength. Li et al. [20] demonstrated that PEDF downregulates Sost/sclerostin, an inhibitor of bone formation, in osteocytes, suggesting a mechanism by which PEDF could promote osteoblastogenesis. Consistently, PEDF engages different intracellular pathways, including ERK/GSK-3β/β-catenin signaling, which regulates genes critical for osteoblast activity and differentiation [21]. Li et al. [22] highlighted that PEDF also activates Wnt signaling via phosphorylation of low-density lipoprotein receptor-related protein 6 (LRP6), thereby enhancing the expression of genes involved in osteoblast maturation and matrix mineralization, thus supporting bone density and quality. In addition, PEDF was interestingly described to also upregulate VEGF expression in mesenchymal stem cells, precursors of osteoblasts [23,24]. Given the pivotal role of VEGF in angiogenesis during bone development and repair, these findings suggest that PEDF supports both osteoblastic differentiation and vascularization, thereby facilitating nutrient and oxygen delivery to developing bone tissue. Beyond its effects on osteoblasts, PEDF inhibits osteoclastogenesis by antagonizing RANKL-mediated signaling, essential for osteoclast survival and function [25]. By coordinating osteoblast and osteoclast behavior, PEDF helps maintain homeostatic bone remodeling. This versatility underscores its central role in orchestrating the mechanisms underlying bone formation, maintenance, and repair.

The reduction in PEDF and VEGF in MRONJ, as detected and validated in our study, was evident in jawbone. This widespread decrease may therefore compromise several interconnected molecular aspects of bone physiology, including osteogenic activity, angiogenesis, and matrix mineralization, which remain essential even in the adult skeleton for preserving bone integrity and supporting repair. Besides its role in bone protection through osteoblast differentiation and vascularization, emerging evidence interestingly implicates PEDF in the regulation of necroptotic signaling by inhibiting necroptosis in hypoxic environments through reducing reactive oxygen species (ROS) levels, modulating mitochondrial dynamics, preventing necrosome formation, and influencing signaling in inflammatory responses [26,27,28,29]. It also regulates cell adhesion molecules and growth factor signaling, thereby affecting angiogenesis and necroptotic susceptibility, as shown in retinal endothelial cells via cadherin-mediated adhesion [30].

Desmosomal cadherins, including desmogleins (DSG) and desmocollins (DSC), are fundamental components of desmosomes that provide intercellular adhesion and mechanical stability in stress-bearing tissues, such as bone. In the bone microenvironment, DSGs cooperate with signaling molecules, other adhesion factors, including N-cadherin, and cytoplasmic proteins to regulate osteoblast connectivity, extracellular matrix adhesion, and cytoskeletal dynamics, all of which are essential for cellular processes and differentiation. As a consequence, desmosomal cadherins are thought to influence bone remodeling, density, and structural integrity.

Desmosomal disruption can in fact compromise bone stability, as seen in multiple myeloma, where malignant cell and osteoblast interaction, mediated by N-cadherin, can impair osteoblast functions and promote osteolysis [31,32,33,34,35].

In addition to their structural roles, desmosomal cadherins also influence intracellular signaling, as demonstrated by DSG3-mediated activation of Src kinases that regulate E-cadherin-dependent adhesion, further impacting osteoblast behavior [32,36].

Consequently, desmosomal cadherins are emerging as potential therapeutic targets for enhancing osteoblast function and counteracting bone loss in disorders like osteoporosis and multiple myeloma. In MRONJ, reduced epithelial adhesion and altered DSG1 expression in the oral epithelium of patients on long-term bisphosphonates suggest that weakened cadherin functions could compromise both osteoblast connectivity and pathway-regulating differentiation [37]. Consistently, our proteomic analysis revealed reduced DSG1 and DSC1 abundance in the MRONJ jawbone, a finding that we confirm and extend by demonstrating, by Western blot, a marked loss of DSG1 and DSC1 in bone tissue from MRONJ patients.

Taken together, these observations further support our depiction of MRONJ as a disorder of structural and signaling integrity, where affected cadherin-mediated adhesion and associated signaling impair bone homeostasis and regeneration. The concomitant downregulation of PEDF and cadherins, observed in MRONJ samples, together with TNFAIP3 inhibition and RIP activation, observed in our cohort of patients, might contribute to the engagement of necroptotic mechanisms within the jaw bone.

This study suggests a multifactorial disruption of bone homeostasis in MRONJ, involving dysfunctional osteogenic signaling, altered cell adhesion, and sustained inflammatory activity. The parallel deregulation of key transcription factors, structural proteins, and inflammatory regulators points to coordinated breakdown in bone remodeling mechanisms, potentially exacerbated by necroptotic processes.

Despite the limited sample size and challenges in obtaining adequate tissue from necrotic lesions representing important constrains, the convergence of miRNAs, proteome and validation data correlates with the reliability of the observed molecular alterations. This provides a novel multi-omics overview of the disorder, whose biochemical and molecular bases are still largely unknown.

Our preliminary findings lay the groundwork for future investigations into the role of osteogenic and inflammatory pathways in MRONJ and might guide the identification of candidate biomarkers and therapeutic targets.

### Study Limitations and Future Perspectives

Collectively, our findings offer novel insights into MRONJ, despite inherent limitations due to the rarity of the condition. The small sample size reflects both the low, though rising, prevalence of MRONJ and the challenges of working with necrotic tissue, which often compromises biomolecular integrity and limits high-quality sample retrieval. The constrained availability of jawbone tissue further complicates comprehensive analyses.

Future research would benefit from larger, ideally multicenter and longitudinal studies to validate the identified biomarkers and to clarify the temporal dynamics of necroptosis and osteogenic signaling dysregulation in MRONJ. Targeted functional studies are also critical to define the specific contribution of Runx1/2, PEDF, desmosomal cadherins, and TNFAIP3 to bone homeostasis and necroptotic pathways.

Moreover, a potential limitation of our study is the difference in age distribution between the MRONJ and control groups, which reflects the epidemiology of the disease, as MRONJ predominantly affects elderly patients exposed to antiresorptive treatments. Aging is known to profoundly impact bone biology, leading to a reduced osteoblast number and activity, altered mesenchymal stem cell differentiation, and an imbalance between bone formation and resorption [38]. In addition, age-related changes in the bone microenvironment include impaired angiogenesis and remodeling of the vascular niche, which can negatively affect bone regeneration and repair capacity. Specifically in the jawbone, advanced age is associated with a reduced proliferative and osteogenic capacity of alveolar osteoblasts, further supporting the presence of baseline biological differences between young and elderly bone tissue. However, several lines of evidence suggest that the molecular alterations identified in our cohort are not simply attributable to physiological bone aging. Indeed, age-related skeletal changes are typically characterized by a gradual decline in bone formation and structural integrity, rather than by the activation of specific pathways such as necroptosis or the coordinated dysregulation of adhesion molecules and angiogenic regulators. In contrast, necroptosis has been increasingly recognized as a mechanism involved in pathological bone conditions, including osteoporosis and inflammatory bone diseases, rather than a hallmark of normal aging per se. Similarly, proteins such as PEDF and VEGF are tightly involved in active bone remodeling and repair processes, and their dysregulation reflects altered osteogenic and angiogenic signaling rather than baseline senescence-related changes. Although preliminary, this study offers an integrated molecular perspective on MRONJ. Importantly, the convergence of miRNA and proteomic data in our study, together with independent validation experiments, supports the presence of a disease-specific molecular signature that is unlikely to be explained solely by chronological age and strengthens the robustness of our findings, while the identification of osteogenesis- and inflammation-related biomarkers opens promising avenues for translational applications in the prevention, management, and treatment of MRONJ [39,40,41].

## 4. Materials and Methods

### 4.1. Patient Enrollment

Patients who had been diagnosed with cancer with bone metastasis, multiple myeloma or osteoporosis were eligible for the study. The study was approved by the Local Ethical Committee of Messina (approval number 36.24 8 March 2024) and was conducted in accordance with the principles of the Declaration of Helsinki.

The inclusion criteria were age ≥ 18 years, a diagnosis of bone metastases from solid tumors or multiple myeloma and current or previous use of zoledronic acid and/or denosumab. Patients with previous radiation in the head and neck area were excluded from the study.

A MRONJ diagnosis had to be performed according to the definition of the Italian Societies of Oral Medicine and Maxillofacial Surgery (the SICMF-SIPMO staging system).

A control group of subjects in good general health conditions, who were surgically treated with the removal of impacted third molars, were used as controls.

#### 4.1.1. Characterization of the Cohort

Demographic data (i.e., age expressed as the mean, sex expressed as a percentage) and the primary cancer type were collected. The suspected drugs related to MRONJ onset, namely, zoledronic acid or denosumab, were recorded. Data concerning concurrent cancer treatments and other medications given for comorbidities were collected. Number of total comorbidities was reported on the basis of the Cumulative Illness Rating Scale (CIRS) available in the patient’s record. Comorbidities were evaluated and categorized as follows: (a) cardiovascular disease, (b) diabetes, (c) chronic kidney disease, and (d) others.

The clinical features of MRONJ patients were characterized to identify the anatomic location of exposed necrotic bone areas, and the MRONJ stage according to the currently adopted classification from the Italian Society of Oral Medicine and Oral Pathology (SIPMO) and potential triggers (oral/dental findings) were recorded [42].

MRONJ diagnosis was based on the following: (a) clinical features of MRONJ (e.g., presence of exposed bone); (b) patients’ medical history, including current or previous treatment with antiresorptive or antiangiogenic agents; and (c) dental radiographs suggestive of MRONJ (Table 5).

#### 4.1.2. Routine Clinical Care

To provide a clear description of the nonsurgical and surgical management of the enrolled MRONJ patients, routine clinical care is described.

Routine procedures at the first examination include oral swab/exposed bone biopsy and pharmacological treatment with systemic antibiotics. The initial treatment was amoxicillin plus clavulanic acid in combination with metronidazole 250 mg; subsequently, patients were switched to targeted antibiotic therapy on the basis of the antibiogram results. The systematic application of this workflow ensures homogeneity in the sample features in terms of antibiotic therapy (empiric vs. targeted therapy). Eight to ten weeks after the initiation of pharmacological treatment in the absence of clinical signs of suppuration, patients underwent a multidisciplinary evaluation with the chief oncologist/hematologist, and the appropriateness of surgery with radical intent was discussed; if no contraindications were present, they underwent MRONJ surgery.

#### 4.1.3. MRONJ Surgery

Surgical options entail conservative bone surgery (sequestrectomy and/or alveoloplasty, resection) and extensive bone surgery with radical intent, which consists of the removal of the radiologically identified necrotic bone determined through bleeding evidence of the surrounding bone. Submarginal resection of the necrotic bone is performed via a piezoelectric device. Suture with interrupted points was provided to secure primary healing to avoid dehiscence. Nerve block of the inferior alveolar and buccal nerves with 3% mepivacaine hydrochloride with adrenaline (1:100,000) was performed.

#### 4.1.4. Surgical Specimen Collection in MRONJ

In MRONJ patients treated with any type of osseous resective surgery, biopsies were taken from the removed bone tissue, including the necrotic area with a rim of adjacent bone. MRONJ samples were selected based on relative morphological homogeneity since osteonecrotic tissue undergoes significant degradation potentially compromising sample quality for biomolecular analysis. The necrotic tissue itself was excluded from the analysis. Collected samples were frozen at −80 °C immediately after surgery and stored at the same temperature until molecular analyses.

#### 4.1.5. Surgical Specimen Collection in Control Group

Bone samples obtained from healthy patients consisted of the surrounding bone removed during osteotomy for lower third molar extraction. Third molar extraction surgeries are carried out after a full-thickness flap with a vertical releasing incision elevation, and ultrasonic osteotomies are performed according to the manufacturer’s instructions using a specific insert for osteotomies. When tooth sectioning is necessary, prophylactic odontotomy is performed with a conventional technique in the extraction process via a Lindemann stainless steel bur mounted on a high-speed straight surgical handpiece. The mucoperiosteal flap is then repositioned, and the surgical wound is closed with interrupted points. Collected samples were frozen at −80 °C immediately after surgery and stored at the same temperature until molecular analyses.

### 4.2. miRNA Analysis

To gain a broad understanding and select a panel of miRNAs involved in bone necrosis, we performed a comprehensive search on PubMed and Science Direct via the keywords “miRNA”, “bone” and “necrosis”. On the basis of the results obtained, we selected 21 miRNAs from 5 articles [12,43,44,45,46]. This selection (miR-30b-5p; miR-34a-3p; miR-181a; mir-125a-3p; miR-135b-5p; miR-155-5p; miR195-5p; miR-204; miR-211; miR-218-5p; miR204-5p; miR-222-3p; miR-325-3p; miR-335-5p; miR-433-3p; miR-497-5p; miR-505-5p; miR-578; miR-628-3p; miR-221-5p; miR-484) was supported by an in silico search of the metabolic pathways potentially involved in necrosis as potential biomarkers to be analyzed in the bone tissues of our patient cohort.

#### 4.2.1. miRNA Extraction

Enriched microRNAs were extracted from frozen stored bone tissue in dry ice at −80 °C via the mirVana™ miRNA Isolation Kit (Ambion, Milan, Italy), and total RNA was also extracted via the Total Nucleic Acid Isolation Kit (Qiagen, Hilden, Germany) following the manufacturer’s protocol. The concentrations of the samples were measured spectrophotometrically via a Bioanalyzer tool (Agilent Technologies, Santa Clara, CA, USA) [47].

##### Reverse Transcriptase Reactions

The reverse transcriptase reaction contained RNA samples, including purified miRNA, 50 nM stem–loop RT primer for each miRNA (RNU6, miR320 and miR-423-5p) purchased from Thermo Fisher (Waltham, MA, USA), 0.25 mM each dNTP, 3.33 U/μL MultiScribe reverse transcriptase (P/N: 4319983, Life Technologies, Segrate, MI, Italy) and 0.25 U/μL RNase inhibitor (P/N: N8080119; Life Technologies, Segrate, MI, Italy). The 7.5 μL reactions were incubated in a thermocycler for 30 min at 16 °C, 30 min at 42 °C and 5 min at 85 °C and then held at 40 °C. All reverse transcriptase reactions, including no-template controls, were run in duplicate [48].

#### 4.2.2. Real-Time PCR

Real-time PCR was performed via a standard TaqMan PCR kit protocol on an Applied Biosystems (Waltham, MA, USA) 7300 instrument. The 10 μL PCRs included 0.67 μL of RT product, TaqMan Universal PCR Master Mix (P/N: 4324018, Life Technologies), and 0.2 μM TaqMan probe. The reactions were incubated in a 96-well plate at 95 °C for 10 min, followed by 40 cycles of 95 °C for 15 s and 60 °C for 1 min. All reactions were run in triplicate. The threshold cycle (CT) is defined as the fractional cycle number at which the fluorescence passes the fixed threshold. TaqMan CT values were converted into absolute copy numbers via a standard curve from miRNA U6. The relative quantitative RQ is expressed in Log2 [48].

#### 4.2.3. Target Prediction Tools

The genes targeted by the predicted miRNAs and the metabolic pathways that are involved were identified by examining the following online databases: miRDB (http://mirdb.org/miRDB/ accessed on 11 July 2024), TargetScan (www.targetscan.org accessed on 12 July 2024), microRNA.org (www.microrna.org accessed on 14 July 2024), PicTar (http://pictar.mdc-berlin.de accessed on 15 July 2024), Gene cards.org and Kyoto Encyclopedia of Genes and Genomes (KEGG) [49,50,51,52]. The enrichment analysis of biological processes related to miRNA targets was carried out with DIA-miRPath v4.0 (https://bio.tools/diana_mirpath_v4.0 accessed on 16 July 2024), and experimentally validated targets retrieved from TargetScan v8.0 were selected [20]. To detect the biological processes associated with the miRNA targets, the gene set from the Reactome Pathway Database was selected, with “gene intersection” used as the merging method and a *p* value cutoff of 0.05 with FDR correction, as previously reported [49,50,51,52,53,54].

### 4.3. Protein Extraction

Frozen samples were mechanically fragmented into coarse pieces and thoroughly washed in cold phosphate-buffered saline (PBS) to reduce blood contamination. Sample homogenization and protein extraction was then carried out in urea 9 M, 4% (*w*/*v*) 3-[(3-cholamidopropyl) dimethylammonio]-1-propane sulfonate (CHAPS), 40 mM Tris, and 65 mM dithiothreitol (DTE) and by using the Tissue Lyser II (Qiagen, Hilden, Germany), at an oscillation frequency of 25 Hz (1500 oscillations per min). Sample disruption was performed through 2 min cycles on the instrument, with 5 min resting intervals at −20 °C, and for a total of 10 cycles. The resulting sample solutions were centrifuged at 20,817× *g*, for 15 min at 4 °C, supernatant-recovered and overnight-precipitated in cold acetone (1:4 *v*/*v*) at −20 °C. Following a 10 min centrifugation step at 20,817× *g* and 4 °C, pellets were dried at room temperature, and resuspended in urea 8 M, 4% (*w*/*v*) CHAPS, and 1% (*w*/*v*) DTE, and further centrifuged at 20,817× *g* for 15 min at 4 °C. Supernatants were recovered, protein estimation was performed according to the Bradford method, and sample aliquots maintained at −80 °C until use.

#### 4.3.1. Proteomics Analysis

Bone protein extracts were separated via two-dimensional electrophoresis (2DE) following the methodology described by Bianchi et al. [55]. For analytical 2DE experiments, 60 μg of protein were mixed with 0.2% (*v*/*v*) pH 3–10 carrier ampholytes and loaded onto isoelectric focusing (IEF) strips (18 cm, pH 3–10 non-linear gradient; Cytiva, formerly GE Healthcare, Marlborough, MA, USA), by using a cathodic cup-loading system and the Ettan IPGphor instrument (Cytiva Marlborough, MA, USA). For preparative 2DE runs, aimed at mass spectrometry (MS) analysis, 500 µg of protein was applied with 2% (*v*/*v*) pH 3–10 carrier ampholytes on the same type of IEF strip used for analytical runs. IEF was performed on an Ettan IPGphor Manifold (Cytiva, Marlborough, MA, USA) under the following conditions at 16 °C: 200 V for 8 h, a gradient from 200 V to 3500 V over 2 h, 3500 V for 2 h, a gradient from 3500 V to 5000 V over 2 h, 5000 V for 3 h, a gradient to 8000 V over 1 h, and finally, 8000 V maintained until a total of 95,000 Vh was reached.

After IEF, the strips were equilibrated, and the second-dimension electrophoresis was performed according to Bianchi et al. [55]. Analytical gels were visualized with ammoniacal silver nitrate staining [56], while preparative gels for MS were stained following the Sinha protocol [57].

Gel images were captured by using the Image Scanner III and processed with the LabScan 6.0 software (GE Healthcare, Chicago, IL, USA). Quantitative analysis of gel spots was performed applying Melanie™ Classic 9 software (SIB Swiss Institute of Bioinformatics, Geneva, Switzerland), with spot volumes expressed as relative percentages (%Vol), representing the ratio of the optical density of each spot to the total spot volume within the same gel (for more details about image analysis: https://2d-gel-analysis.com/). %Vols were then exported for statistical analysis using R.

Proteomic comparisons between mandibular bone specimens from patients affected by osteonecrosis and controls, from apparently healthy donors, were conducted on %Vols by applying Bayesian analysis with the limma R package (v.4.4) [58]. Individual spots corresponding to statistically significant differences (FDR ≤ 0.05), which showed a relevant fold change (FC) ≥ 2, were checked for reproducibility across biological replicas and the accepted ones visualized in a heatmap, with clustering based on Ward’s method for Euclidean distances. A principal component analysis (PCA) was also applied to the %Vol values of significantly differing spots to examine variance and covariance occurring among their corresponding samples. Statistical analyses and related figures were generated by applying the R software (R Core Team, Vienna, Austria, available at https://www.R-project.org; accessed 1 September 2024).

#### 4.3.2. In-Gel Digestion and LC–MS/MS Analysis

Differential spots of statistical significance were excised manually from MS-preparative gels and subjected to destaining [59]. The spots were then partially dehydrated in a solution of 5 mM ammonium bicarbonate and 50% (*v*/*v*) acetonitrile, followed by complete dehydration in 100% acetonitrile (ACN). The protein spots were processed by in-gel digestion via trypsin (Promega, Madison, WI, USA), as previously described [57]. Peptide mixtures were extracted twice from the gel with acetonitrile and resuspended in 0.2% formic acid. The samples were then analyzed via liquid chromatography–tandem mass spectrometry (LC–MS/MS) via an Orbitrap Exploris 240 mass spectrometer equipped with a Vanquish Neo UHPLC (both Thermo Fisher Scientific, Waltham, MA, USA). LC–MS/MS analysis was performed as published previously with slight modifications [60,61]. Briefly, buffer A (0.1% (*v*/*v*) formic acid in water) and buffer B (0.1% (*v*/*v*) formic acid in 80% acetonitrile) were used to separate peptides in a 60 min gradient as follows: 2% buffer B for 3 min, 30% buffer B from 3 to 33 min, 50% buffer B from 33 to 48 min, 90% buffer B from 48 to 53 min, 95% buffer B from 53 to 60 min for column washing and equilibration. The system was operated in data-dependent acquisition (DDA) mode, and the top 20 most abundant ions were selected on each MS spectrum for further isolation over a scan range of 300–1800 *m*/*z* with an Orbitrap mass resolution of 120,000. Tandem MS/MS spectra were acquired with a resolution of 15,000 by higher-energy collisional dissociation (HCD) fragmentation of the top 20 most intense peaks. The HCD was set with an NCE of 30%, and the dynamic exclusion time was set as 25 s.

MaxQuant (v2.6.7.0—https://www.maxquant.org; [62]) was used for protein identification via the fasta file of the human reference proteome (UniProt UP000005640). Searches were performed with tryptic specifications and default settings for mass tolerances for MS and MS/MS spectra. Fixed modifications included Cys carbamidomethylation, whereas Met oxidation and N-terminal acetylation were variable modifications. The minimum peptide length was set to seven amino acids, and the false discovery rate (FDR) for proteins and peptide-spectrum matches (PSM) was set to 1%. Common contaminants, peptides identified only by site modification and reverse peptides were excluded prior to further analysis. The software search was restricted to SwissProt entries and high-confidence proteins were selected based on the identification of at least 6 unique peptides. High MS/MS spectral counts (PSMs) and iBAQ (intensity-based absolute quantification) values were also used to prioritize the obtained identifications.

### 4.4. Western Blot Analysis

MRONJ and healthy jawbone samples were homogenized in liquid nitrogen with a pre-cooled mortar and pestle, yielding a fine powder employed for protein extraction, using a lysis buffer (RIPA buffer: 10 mM Tris-HCl pH 7.4, 1 mM EDTA, 1 mM EGTA, 1% Nonidet P-40, 1% Triton X-100, 150 mM NaCl; supplemented with protease inhibitors: aprotinin, leupeptin, pepstatin A, phenylmethylsulfonyl fluoride). All procedures were performed under cold-chain conditions. The collected lysates were centrifuged at 15,000× *g* for 15 min at 4 °C. Supernatants were recovered, and protein concentrations were determined using the Bradford protein assay (Bio-Rad Laboratories, Hercules, CA, USA), with bovine serum albumin as the standard. Absorbance was measured spectrophotometrically at 595 nm. Protein samples were diluted 1:1 in Laemmli SDS sample buffer, prepared by mixing 2-mercaptoethanol with 2× Laemmli Sample Buffer (Bio-Rad) at a ratio of 1:19. The reducing agent 2-mercaptoethanol cleaves disulfide bonds, an effect enhanced by heating the samples to 95 °C for 5 min, thus promoting protein denaturation and preventing secondary structure interference during migration. Proteins were separated on 7.5% or 10% polyacrylamide gels containing SDS, run in electrophoresis buffer at a constant current of 200 mA. Subsequently, proteins were transferred onto polyvinylidene fluoride (PVDF) membranes at a constant current of 100 mA for one hour in transfer buffer. Membranes were blocked for one hour in 5% nonfat dry milk (NFDM) to prevent nonspecific binding. After three washes with TBS-Tween 0.1%, membranes were incubated overnight at 4 °C with primary antibodies diluted in TBS-Tween 0.1%, targeting GAPDH (Santa Cruz Biotechnology, Dallas, TX, USA), TNFAIP3 (Santa Cruz Biotechnology, Dallas, TX, USA), RUNX2 (Santa Cruz Biotechnology, Dallas, TX, USA), RUNX1 (Santa Cruz Biotechnology, Dallas, TX, USA), DSG1 (Santa Cruz Biotechnology, Dallas, TX, USA), DSC1 (Santa Cruz Biotechnology, Dallas, TX, USA), RIP3 (Santa Cruz Biotechnology, Dallas, TX, USA), PEDF (Santa Cruz Biotechnology, Dallas, TX, USA) and VEGF (Santa Cruz Biotechnology, Dallas, TX, USA). Following incubation, unbound primary antibodies were removed by three additional washes with TBS-Tween 0.1%. Membranes were then incubated for one hour at room temperature with horseradish peroxidase (HRP)-conjugated Goat Anti-Rabbit IgG secondary antibody (Sigma-Aldrich, St. Louis, MO, USA) diluted in 0.15% TBS-Tween containing 5% NFDM. After three washes with 0.15% TBS-Tween, protein bands were visualized using Amersham™ ECL Prime Western blotting Detection Reagent (Cytiva, Marlborough, MA, USA). Quantification was performed by densitometric analysis with the LICOR imaging system, and results were expressed as relative intensity normalized to GAPDH, used as an endogenous control.

### 4.5. Statistical Analysis

A descriptive analysis of the demographic and clinical characteristics of the patients was performed. Categorical variables were expressed as percentage frequencies, while continuous variables were reported as medians and interquartile ranges (IQR). The Kolmogorov–Smirnov test was used to assess the normality of data distribution. Since some variables were not normally distributed, a non-parametric approach was adopted. A *p* ≤ 0.05 was considered significant. Statistical analysis was performed via Stata (18.0).

## Figures and Tables

**Table 1:** Profile of MIRNA. Reported values are considered upregulated when ≥2.

MiRNA	Variable: Median (IQR)
**Upregulated miRNAs:**	
MiR-30b-5p	2.35 (2.17–2.60)
MiR-34a-3p	2.45 (2.3–2.62)
MiR-181a	2.55 (2.47–2.82)
Mir-125a-3p	2.50 (2.37–2.62)
MiR-135b-5p	2.80 (2.65–2.85)
MiR-155-5p	2.75 (2.60–2.82)
MiR-195-5p	2.55 (2.37–2.72)
MiR-204	2.34 (2.24–2.51)
MiR-211	2.32 (2.14–2.57)
MiR-218-5p	2.80 (2.65–2.09)
MiR-204-5p	2.40 (2.27–2.5)
MiR-222-3p	2.50 (2.27–2.62)
MiR-325-3p	2.21 (2.04–2.34)
MiR-335-5p	2.55 (2.27–2.72)
MiR-433-3p	2.30 (2.2–2.75)
MiR-497-5p	2.1 (2.02–2.2)
MiR-505-5p	2.08 (2.01–2.22)
MiR-578	2.21 (2.06–2.57)
MiR-628-3p	2.30 (2.09–2.5)
**Downregulated miRNAs:**	
MiR-221-5p	1.60 (1.40–1.80)
MiR-484	1.40 (1.15–1.65)

**Table 2:** GO analysis of targets of upregulated miRNAs found in ONJ patients vs. controls.

Category	Term	Count	*p*-Value	FDR
Biological process	GO:0048523—negative regulation of transcription	39	9.25 × 10^−12^	1.25 × 10^−8^
	GO:0016055—Wnt signaling pathway	81	7.661 × 10^−11^	8.341 × 10^−9^
	GO:0023051—damaged DNA binding	39	6.261 × 10^−12^	5.273 × 10^−9^
Molecular function	GO:0140297—DNA binding	20	8.224 × 10^−11^	4.47 × 10^−9^
	GO:0031625—ubiquitin protein ligase binding	18	7.66 × 10^−10^	8.347 × 10^−6^
	GO:0008134—transcription factor binding	20	2.56 × 10^−7^	5.83 × 10^−5^
Cellular components	GO:0043231—intracellular membrane-bounded organelle	22	2.518 × 10^−7^	2.62 × 10^−6^
	GO:0031981—Nuclear lumen	23	2.518 × 10^−6^	2.21 × 10^−9^
	GO:0019005—ubiquitin ligase complex	22	0.00000181	2.17 × 10^−7^
	GO:0031090—organelle membrane	23	2.11 × 10^−8^	2.13 × 10^−7^

**Table 3:** KEGG pathway analysis of targets of upregulated miRNAs found in ONJ patients vs. controls.

KEGG Pathways	Count	*p*-Value	FDR
hsa04360: HIF-1 signaling pathway	18	3.47 × 10^−8^	3.95 × 10^−7^
hsa04010: PI3K-Akt signaling pathway	19	1.47 × 10^−7^	1.39 × 10^−6^
hsa04068: FoxO signaling pathway	20	2.86 × 10^−7^	1.24 × 10^−7^

**Table 4:** LC-MS/MS protein-spot identifications.

Spot Number	Majority Protein IDs	Protein Names	Gene Names	Unique Peptides	iBAQ	MS/MS Count
2	Q02413	Desmoglein-1	*DSG1*	8	167320	6
3	P02675	Fibrinogen beta chain	*FGB*	23	59372000	38
	P25705	ATP synthase subunit alpha, mitochondrial	*ATP5F1A*	11	7589800	12
	P06576	ATP synthase subunit beta, mitochondrial	*ATP5F1B*	10	5483300	11
	P07196	Neurofilament light polypeptide	*NEFL*	9	4218300	11
	P11142	Heat shock cognate 71 kDa protein	*HSPA8*	9	2100300	13
	P68871	Hemoglobin subunit beta	*HBB*	8	19580000	10
	Q08554	Desmocollin-1	*DSC1*	8	1637000	13
	P17600	Synapsin-1	*SYN1*	7	584520	8
	P21796	Voltage-dependent anion-selective channel protein 1	*VDAC1*	6	3753500	7
	P23528	Cofilin-1	*CFL1*	6	7414500	7
	Q02413	Desmoglein-1	*DSG1*	6	730300	9
	Q16352	Alpha-internexin	*INA*	6	1221000	6
4	P36955	Pigment epithelium-derived factor	*SERPINF1*	7	2627100	8
5	P36955	Pigment epithelium-derived factor	*SERPINF1*	11	8461000	13
	P81605	Dermcidin	*DCD*	7	96472000	11
	P15924	Desmoplakin	*DSP*	6	62204	7
	P02675	Fibrinogen beta chain	*FGB*	6	475000	6

**Table 5:** Clinical characteristics of study participants.

	MRONJ n = 10	Controls n = 6
**Mean age (years)**	74.6	35.3
**Male**	3 (30.00%)	5 (83.33%)
**Female**	7 (70.00%)	1(16.67%)
**Administered antiresorptive drug**		
Denosumab 120 mg (subcutaneous)	3 (30.00%)	
Denosumab 60 mg (subcutaneous)	1 (10.00%)	
Zoledronate (e.v)	4 (40.00%)	
Alendronate (per os)	2 (20.00%)	
**Primary Pathology**	N. (%)	
Breast cancer	5 (50.00%)	
Prostate cancer	2 (20.00%)	
Multiple myeloma	1 (10.00%)	
Osteoporosis	2 (20.00%)	
**Cumulative Illness Rating Scale (CIRS)**		
N. of total comorbidities (Mean)	4	
**Comorbidities**		
Cardiovascular disease	3 (30.00%)	
Diabetes	1 (10.00%)	
Chronic kidney disease	2 (20.00%)	
Others	2 (20.00%)	

## Data Availability

The datasets used and/or analyzed during the current study are available from the corresponding author upon reasonable request.

## Supplementary Materials

The following supporting information can be downloaded at: https://www.mdpi.com/article/10.3390/ijms27115141/s1.

## Abbreviations

MRONJ	Medication-related osteonecrosis of the jaw
PEDF	pigment epithelium-derived factor
VEGF	vascular endothelial growth factor
DSG	desmoglein
DSC	desmocollin
RIPK	receptor-interacting protein kinase
RunxI	Runt-related transcription factor 1
Runx2	Runt-related transcription factor 2
NAFT5	Nuclear Factor of Activated T-cells
TNFAIP3	Tumor Necrosis Factor Alpha-Induced Protein 3
