# Supplementary material for: Proteome and miRNAs Expression in Medication-Related Osteonecrosis of the Jaw
Source: Int J Mol Sci. 2026 Jun 5;27(11):5141. doi: 10.3390/ijms27115141 (PMC13257934; doi:10.3390/ijms27115141)
Supplement: Supplementary file 1 [file ijms-27-05141-s001.zip › ijms-4293345-supplementary.pdf]

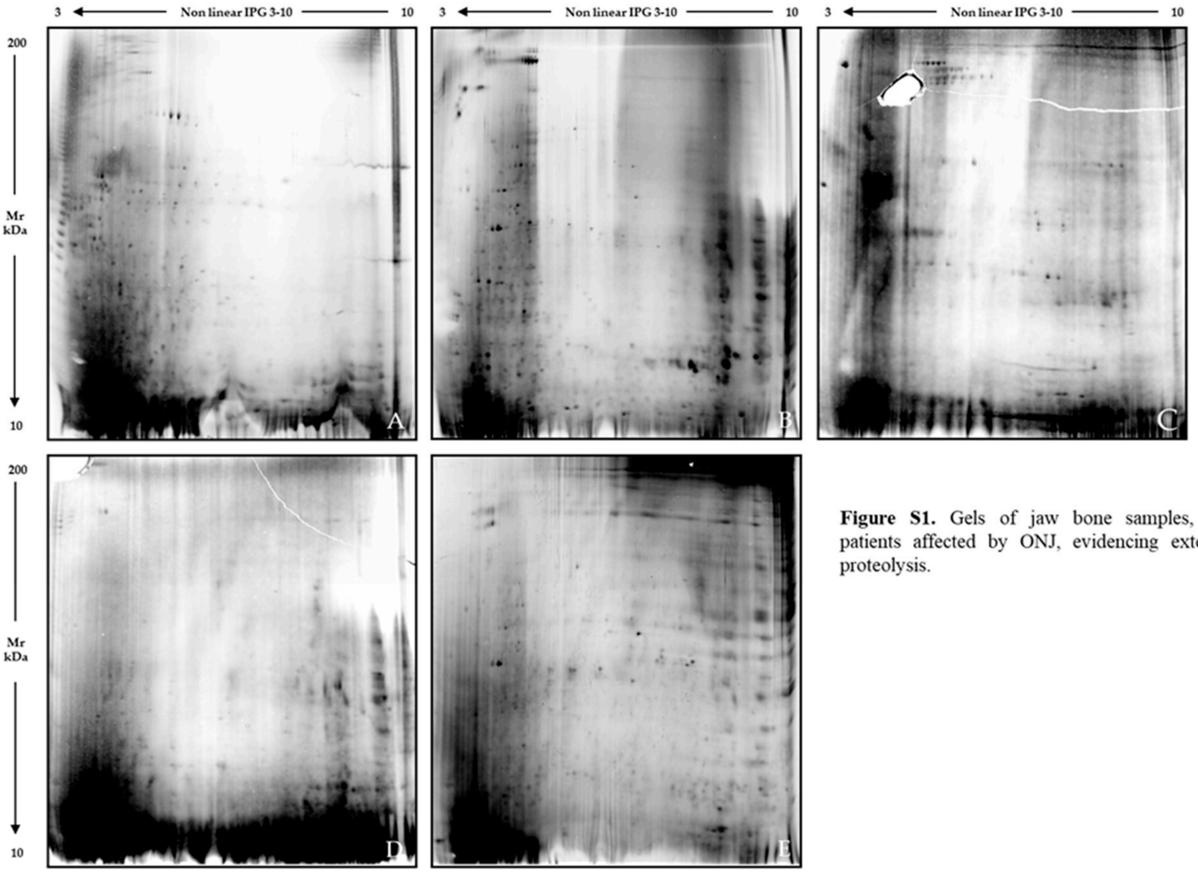

Figure S1. Gels of jaw bone samples, from patients affected by ONJ, evidencing extensive proteolysis.
